# Quantification of Tryptophan and NAD ^+^ Proton Magnetization Exchange With Water Using Downfield ^1^H MRS in the Human Brain at 7 T

**DOI:** 10.1002/mrm.70134

**Published:** 2025-10-14

**Authors:** Sophia Swago, Neil E. Wilson, Mark A. Elliott, Ravinder Reddy, Walter R. Witschey, Ravi Prakash Reddy Nanga

**Affiliations:** ^1^ Department of Bioengineering, School of Engineering and Applied Sciences University of Pennsylvania Philadelphia Pennsylvania USA; ^2^ Department of Radiology, Perelman School of Medicine University of Pennsylvania Philadelphia Pennsylvania USA

**Keywords:** brain, chemical exchange, cross‐relaxation, downfield spectroscopy, magnetic resonance spectroscopy, spectral excitation, T_1_ relaxation time

## Abstract

**Purpose:**

The purpose of this study was to investigate the effect of magnetization exchange on the measurement of tryptophan and NAD^+^ T_1_ relaxation times and to determine the magnetization exchange rates with a two‐spin system model using downfield ^1^H MRS spectroscopy at 7 T in human brain.

**Methods:**

We collected downfield ^1^H MRS spectra in the human brain of eight healthy volunteers using a spectrally selective single‐slice sequence (excitation window: 9.7 ± 2 ppm) at 7 T. Alternating selective and broadband saturation recovery experiments were performed to probe magnetization transfer‐dependent changes in T_1_ recovery. The apparent T_1_ was modeled independently for each experiment for the tryptophan resonance at 10.1 ppm and for the NAD^+^ resonances at 9.3, 9.1, and 8.9 ppm. The magnetization exchange rate was modeled explicitly using a two‐spin model to fit both experiments simultaneously.

**Results:**

The apparent T_1_ relaxation times measured from broadband saturation recovery experiments were significantly longer for each resonance compared to those measured from selective saturation (*p* < 0.001). The ratio of broadband to selective T_1_ was significantly larger for tryptophan than NAD^+^ (TRP: 19 ± 6 vs. NAD^+^: 9 ± 3; *p* < 0.01). Using the two‐spin model, we modeled the T_1_ of each resonance (ms): T_1,TRP_ = 622.3 ± 405.5; T_1,NAD,H2_ = 924.9 ± 233.7; T_1,NAD,H6_ = 1800.0 ± 985.5; T_1,NAD,H4_ = 2057.3 ± 573.5. The chemical exchange rate of tryptophan was 12.3 ± 3.1 Hz; the cross‐relaxation rates of NAD^+^ were (Hz): *σ*_NAD,H2_ = 5.7 ± 1.6, *σ*_NAD,H6_ = 3.3 ± 0.8, and *σ*_NAD,H4_ = 3.0 ± 1.5. The tryptophan exchange rate was significantly faster than the rates for NAD^+^ (*p* < 0.001).

**Conclusion:**

Tryptophan chemical exchange and NAD^+^ cross‐relaxation with water can be quantified in vivo in human brain at 7 T using downfield spectroscopy.

## 1 Introduction

Proton magnetic resonance spectroscopy (^1^H MRS) provides a method to non‐invasively study cellular metabolism in vivo. In the brain, there are a large number of metabolites that can be detected with conventional MRS techniques, including N‐acetylaspartate, choline, myoinositol, creatine, lactate, glutamine, and glutamate; these act as various biomarkers of neuronal health, cell membrane synthesis, energy metabolism, and neurotransmission [1]. These metabolites all have resonances in the upfield portion of the proton magnetic resonance spectrum (< 4.7 ppm). However, there are an emerging number of metabolites that resonate downfield of water (> 4.7 ppm) that are of great interest [2, 3, 4]. The detection of these downfield metabolites is complicated by the typical use of water suppression in ^1^H MRS experiments. Many downfield metabolites undergo magnetization exchange with water via cross‐relaxation of non‐labile protons or through chemical exchange of labile protons, making these resonances difficult to detect and prolonging their T_1_ relaxation times under water‐suppressed conditions [2, 5, 6, 7]. This has led to the development and use of techniques that do not use water suppression, such as metabolite cycling [5, 8] and spectrally selective excitation [9, 10] in order to study downfield metabolite resonances.

Understanding magnetization exchange is not only important for the development and optimization of downfield spectroscopy sequences but may also provide insight into interactions between metabolites and the cellular microenvironment. Magnetization exchange has primarily been investigated for both upfield and downfield metabolites using magnetization transfer (MT) and chemical exchange saturation transfer (CEST) experiments. MT experiments investigate magnetization exchange between immobile and mobile protons, such as between bound and free water, by employing off‐resonance pre‐irradiation [11, 12]. MT effects have also been shown to exist for several upfield metabolite resonances, such as those arising from total creatine and lactate methyl protons, as a result of cross‐relaxation with water [13, 14, 15]. The binding of lactate to macromolecules may contribute to the MT effect seen for the lactate methyl protons observed in the tumor environment [16], while the MT effect for creatine has been suggested to reflect interactions with proteins or other macromolecules, but not creatine kinase [17]. The CEST experiment relies on the prolonged saturation of metabolite protons which then exchange with water, resulting in a reduced measured water signal. The CEST effect has been investigated using the downfield amine groups of glutamate and creatine [18, 19], as well as the hydroxyl groups of glycosaminoglycan and glycogen [20, 21]. The downfield amide groups of proteins and peptides have also been used as a source of CEST contrast in amide proton transfer (APT) imaging [22]. CEST has emerged as a method that allows high‐resolution imaging of metabolite and protein content, as well as pH‐related changes, in several brain pathologies, including tumors [23, 24], epilepsy [25], and multiple sclerosis [26].

MacMillan et al. measured magnetization rates of downfield resonances in the 6–9 ppm region in the human brain [5] and of upfield the creatine resonances in calf muscle at 3 T [27]. Our group previously quantified the cross‐relaxation of resonances in the 8–8.5 ppm range in human calf muscle at 7 T [7], while Shemesh et al. observed an enhancement of longitudinal relaxation in the resonances in the 7–9 ppm region in excised mouse brain at 9.4 T [6]. However, there are additional downfield metabolites for which magnetization exchange has not been investigated, including nicotinamide adenine dinucleotide (NAD^+^) and tryptophan (TRP). NAD^+^ has three downfield resonances at 8.9, 9.1, and 9.3 ppm, and is central to oxidative phosphorylation, as well as in processes of DNA repair and cell signaling [28, 29]. Tryptophan has a detectable downfield proton resonance at 10.1 ppm and is a key precursor for NAD^+^, serotonin, and melatonin in the brain [30, 31]. Though de Graaf et al. observed an increase in NAD^+^ T_1_ relaxation time using nonselective inversion compared to selective inversion metabolites in rat brain in situ [2] and Nanga et al. observed significant tryptophan signal loss upon application of water suppression in the human brain [4], the effect of magnetization transfer on these metabolites has not yet been quantified in humans in vivo.

The current study quantifies the effect of magnetization exchange between water and the metabolites NAD^+^ and tryptophan using downfield MRS in the human brain in a whole‐body 7 T scanner. We utilized spectrally selective excitation and performed two saturation recovery experiments, one using selective saturation and one using broadband saturation. We compared the apparent T_1_ relaxation of NAD^+^ and tryptophan from the two saturation recovery experiments and additionally quantified their rates of magnetization exchange with water through either cross‐relaxation or through chemical exchange using a two‐spin model of longitudinal relaxation.

## 2 Methods

### 2.1 Participants

Eight healthy volunteers with no history of neurological or psychiatric disease were scanned in accordance with local Institutional Review Board regulations. Prior to the MRI scans, all subjects gave written informed consent. Participants were between the ages of 23–42 years old (5 male and 3 female).

### 2.2 In Vivo Magnetic Resonance Spectroscopy

**FIGURE 1:**
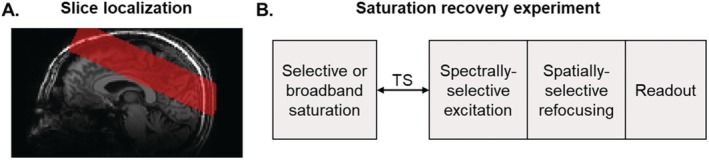
(A) Representative localization of an axially obliqued slice overlaid on a T_1_‐weighted image. (B) Block schematic of the single‐slice spectrally selective sequence, with broadband or selective saturation at some delay TS before excitation.

Downfield ^1^H MRS spectra were collected at 7 T (Magnetom Terra, Siemens Healthcare, Erlangen, Germany) using a 32‐channel head coil (Nova Medical, Wilmington, MA, USA). Data were acquired using a spectrally selective downfield spectroscopy sequence consisting of a 90° sinc pulse (bandwidth (FWHM) = 2 ppm, pulse center = 9.7 ppm) followed by a low bandwidth 180° spatial refocusing SLR pulse for slice localization [10]. An MPRAGE scan was acquired (TR = 2300 ms, TE = 3.49 ms, FOV = 180 × 240 × 160 mm, resolution = 2 × 2 × 2 mm) to localize the slice. A 40 mm thick slice was selected to measure signals from the micromolar concentration metabolites as previously reported [10, 32]. The slice was oriented axially in the brain and obliqued to avoid B_0_ inhomogeneities near the frontal sinus (Figure 1).

Two saturation recovery experiments were carried out for each subject. In the first, a selective 90° sinc pulse (bandwidth = 600 Hz, pulse center = 9.7 ppm) preceded the excitation by a saturation delay time (TS) (TR = 1200 ms, TE = 13 ms, NEX = 64). In the second, a broadband 90° sinc pulse centered at 7.2 ppm (bandwidth: 4000 Hz) saturated both downfield metabolites and water (TR = 6.5 s, TE = 13 ms, NEX = 64). Selective saturation delay times were TS = 25, 50, 300, 600 ms; broadband saturation delay times were TS = 500, 1000, 2000, 4000 ms. A downfield spectrum with no saturation (TR = 2000 ms, TE = 13 ms, NEX = 128) was collected as a measurement of the equilibrium magnetization, which for T_1_ and cross‐relaxation fitting purposes was assigned an arbitrarily long TS of 10 s. A water reference spectrum was also collected using the same spectrally selective sequence (bandwidth = 2 ppm, pulse center = 4.7 ppm, TR = 10 s, TE = 13 ms, NEX = 16) and used to determine coil channel coefficients for offline reconstruction. Manual shimming was performed over the localized slice for each subject, resulting in an average water FWHM of 22.1 ± 2.1 Hz. The total scan time for the entire acquisition protocol was approximately 38 min. The MRS parameters are summarized in Table S1, following the minimum reporting standards for in vivo MRS [33].

### 2.3 MRS Data Processing and Peak Fitting

Data were processed offline from raw complex time‐domain signal files using custom software written in MATLAB as previously described in [32] (Matlab, The Mathworks, Natick, MA, USA) (https://github.com/markymarkymark/SpecTickle). No frequency or phase correction was done on individual transients before averaging due to the low concentration of the metabolites and the lack of a water signal within the metabolite spectra. After averaging the transients for each channel, coil channel combination was done using the water reference scan to calculate appropriate channel weightings. Channel‐wise frequency corrections were applied, and a similarity metric was used to determine channels that could not be corrected, which were then removed [10]. Finally, all data had 5 Hz exponential line broadening applied. Data from the non‐saturation scan were fit in the time domain using Hankel singular value decomposition with 60 components [34, 35]. A single component was assigned to each of the NAD^+^ resonances at 8.9, 9.1, and 9.3 ppm and to the tryptophan resonance at 10.1 ppm. All other components were assigned to the baseline for removal. No other baseline corrections were performed. The components assigned to the NAD^+^ and tryptophan resonances constituted a basis set of fixed frequencies and linewidths that was then used to fit the remaining saturation recovery spectra via complex linear regression.

### 2.4 Relaxation Modeling

The resulting peak amplitudes were fit using two relaxation models. Model 1 fit the selective and broadband saturation recovery curves each with a 3‐parameter exponential model in order to calculate the apparent T_1_ relaxation time for each condition:

(1) $$S\left(\mathrm{TS}\right)=M_{0}\left(1-k*e^{-\frac{\mathrm{TS}}{T_{1}}}\right)$$

where *M*_0_ is the equilibrium magnetization and k is the saturation efficiency (range [0 = no saturation, 1 = complete saturation]).

In Model 2, the rate of magnetization exchange was measured by fitting the two saturation recovery curves simultaneously using modified Bloch/Solomon equations for a two‐spin system [36, 37]:

(2) $$\begin{array}{cc} \text{Spin}\;A:\frac{dM_{z,A}\left(t\right)}{\mathrm{dt}} & =-\frac{\left[M_{z,A}\left(t\right)-M_{z,A,0}\right]}{T_{1,A}}-\sigma_{\mathrm{AB}}\left(M_{z,A}\left(t\right)-M_{z,A,0}\right)\\ & \;+\sigma_{\mathrm{BA}}\left(M_{z,B}\left(t\right)-M_{z,B,0}\right) \end{array}$$

(3) $$\begin{array}{cc} \text{Spin}\;B:\frac{dM_{z,B}\left(t\right)}{\mathrm{dt}} & =-\frac{\left[M_{z,B}\left(t\right)-M_{z,B,0}\right]}{T_{1,B}}-\sigma_{\mathrm{BA}}\left(M_{z,B}\left(t\right)-M_{z,B,0}\right)\\ & \;+\sigma_{\mathrm{AB}}\left(M_{z,A}\left(t\right)-M_{z,A,0}\right) \end{array}$$

where spin A is the metabolite proton and spin B is the water proton. *M*_*z*,*X*_(*t*) is the longitudinal magnetization at time *t* of spin X and *M*_*z*,*X*,0_ is the equilibrium magnetization of spin X, either the metabolite or water. The forward and reverse magnetization exchange rates are *σ*_AB_ and *σ*_BA_, respectively, and follow the relationship *σ*_*AB*_ = *σ*_*BA*_(*M*_*z*,*B*,0_)/(*M*_*z*,*A*,0_). For the selective saturation recovery experiment, the initial conditions are: *M*_*z*,*A*_(0) = *M*_*z*,*A*,0_(1 − *k*_sel_) and *M*_*z*,*B*_(0) = *M*_*z*,*B*,0_, where *k*_sel_ is the efficiency of the selective saturation determined from the three‐parameter fit of the selective saturation recovery curve; for the nonselective saturation recovery experiment, the initial conditions are: *M*_*z*,*A*_(0) = *M*_*z*,*A*,0_(1 − *k*_broad_) and *M*_*z*,*B*_(0) = *M*_*z*,*B*,0_(1 − *k*_broad_), where *k*_broad_ is the efficiency of the broadband saturation determined from the three‐parameter fit of the broadband saturation recovery curve. *M*_*z*,*B*,0_ was calculated from the water reference scan. The model was fit in Python 3.6.13 using *solve_ivp* from the SciPy module (v 1.5.2) [38]. The objective function that was minimized using the basin‐hopping algorithm with least squares in the Scipy module *optimize* was the sum of squared residuals between the selective and broadband measured values and estimated values calculated using Equations (2) and (3):

(4) $$\begin{array}{c} \min_{x}\;{\left\|s_{m}-s_{p}\right\|}_{\mathrm{sel}}+{\left\|s_{m}-s_{p}\right\|}_{\text{broad}} \end{array}$$

where *x* is the model unknowns, which consist of T_1A_ (longitudinal relaxation corrected for magnetization exchange effects) and the magnetization exchange rate for each metabolite *σ*_met_ = *σ*_AB_. We made the following assumptions: T_1_ of water = 1800 ms [39] and the ratio of metabolite to water equilibrium magnetization *M*_*z*,*B*,0_/*M*_*z*,*A*,0_ = 0.3 mM/(2 × 55) M, where 0.3 mM is the average concentration of NAD^+^ in brain and 55 M is the average water concentration, corrected for two protons contributing to the water signal [32, 40, 41].

### 2.5 Statistical Analysis

All statistical tests were performed in R v4.3.2 (R Foundation for Statistical Computing, Vienna, Austria). A *t*–test was performed between the measured selective and broadband apparent T_1_ relaxation times (Model 1) for each resonance (tryptophan at 10.1 ppm, NAD^+^ H2 at 9.3 ppm, NAD^+^ H6 at 9.1 ppm, and NAD^+^ H4 at 8.9 ppm). Analysis of variance (ANOVA) was used to compare the ratio of broadband to selective apparent T_1_ times between the resonances. Additionally, ANOVA was used to compare the corrected T_1_ relaxation time and the magnetization exchange rates (Model 2) between the four resonances (tryptophan, NAD^+^ H2, NAD^+^ H6, and NAD^+^ H4). After each ANOVA test, post hoc Tukey HSD tests were performed to correct for multiple comparisons.

## 3 Results

**FIGURE 2:**
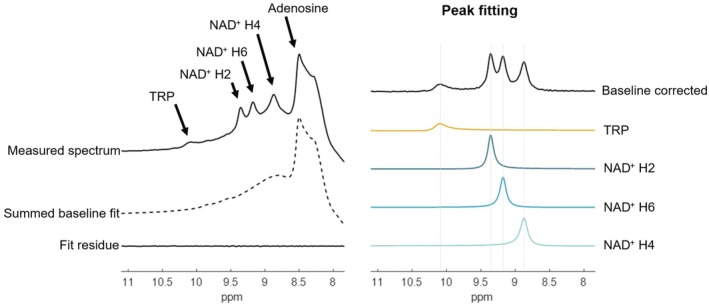
Left: Representative downfield spectrum with tryptophan (TRP) resonating at 10.1 ppm and NAD^+^ proton resonances at 8.9, 9.1, and 9.3 ppm, with summed baseline fit and total fit residual; adenosine resonances are partially observed at the limit of the excitation band. Right: The baseline‐corrected measured spectrum and HSVD fit components assigned to tryptophan and the three NAD^+^ peaks.

Figure 2A shows a representative downfield spectrum (no saturation) with resonances originating from tryptophan and NAD^+^. A single complex Lorentzian was fit to each peak during the HSVD fitting procedure (Figure 2B). Representative spectra at all saturation times are shown in Figure S1. Tryptophan peak fitting failed in one subject due to low SNR. Additionally, in one subject the H6 NAD^+^, and in two subjects the H4 NAD^+^ proton, were omitted due to poor peak fitting quality. The linewidths of the peaks were: TRP: 54 ± 7 Hz, NAD^+^ H2: 28 ± 2 Hz, NAD^+^ H6: 32 ± 2 Hz, and NAD^+^ H4: 38 ± 8 Hz. The SNR of the downfield spectra, calculated as the amplitude of the NAD^+^ H2 resonance in the no saturation scan divided by the standard deviation of a signal‐free region on the upfield side of the spectrum, was 27.4 ± 4.9.

### 3.1 Model 1

**FIGURE 3:**
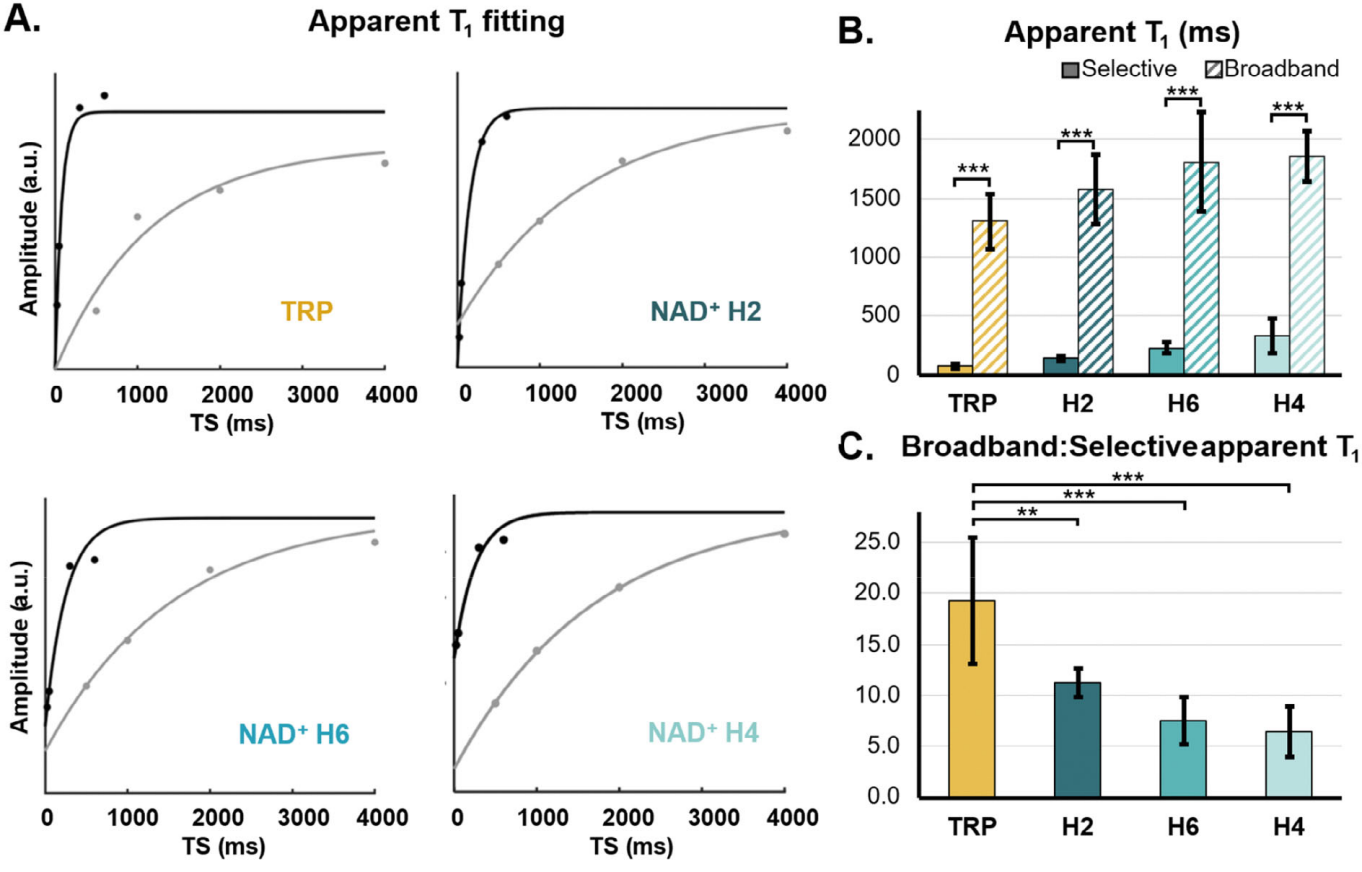
(A) Representative apparent T_1_ recovery curves (Model 1) of tryptophan (TRP) and the NAD^+^ H2, H6, and H4 resonances after separate fitting of selective (black) and broadband saturation (gray). The last saturation time (TS = 10 s assigned to the M_0_ scan) is not shown for illustrative purposes. (B) Mean apparent T_1_ of NAD^+^ resonances measured using selective and broadband saturation recovery. For each resonance, the apparent T_1_ under broadband saturation was longer than under selective saturation. (C) The ratio of the apparent T_1_ under broadband saturation to the apparent T_1_ under selective saturation. *p < 0.05, **p < 0.01, ***p < 0.001.

For all four peaks, we found that the signal was significantly attenuated under the broadband saturation condition in comparison to the spectrally selective saturation and a significant increase in the apparent T_1_ (*p* < 0.001) (Figure 3A). The smallest *R* ^2^ across all apparent T_1_ fits and across all subjects and peaks was 0.96, and the average *R* ^2^ was 0.98 ± 0.02. The mean apparent T_1_ relaxation times and saturation efficiencies for each peak are found in Table S2. We found the broadband apparent T_1_ relaxation time of tryptophan was, on average, 19 ± 6 times longer than the selective apparent T_1_ relaxation time. For NAD^+^, the broadband apparent T_1_ relaxation time were an average of 11 ± 1, 8 ± 2, and 6 ± 2 times longer than the selective apparent T_1_ relaxation times for the H2, H6, and H4 protons of NAD^+^, respectively (Figure 3B). After ANOVA showed there were significant differences in the ratio of broadband to selective apparent T_1_ times between tryptophan, and the three NAD^+^ resonances (*p* < 0.001), the Tukey HSD post hoc test revealed that the increase in apparent T_1_ relaxation time for tryptophan was significantly larger compared to all of the NAD^+^ resonances (TRP vs. NAD^+^ H2: *p* = 0.001; TRP vs. NAD^+^ H6: *p* < 0.001, TRP vs. NAD^+^ H4: *p* < 0.001) (Figure 3C).

### 3.2 Model 2

**FIGURE 4:**
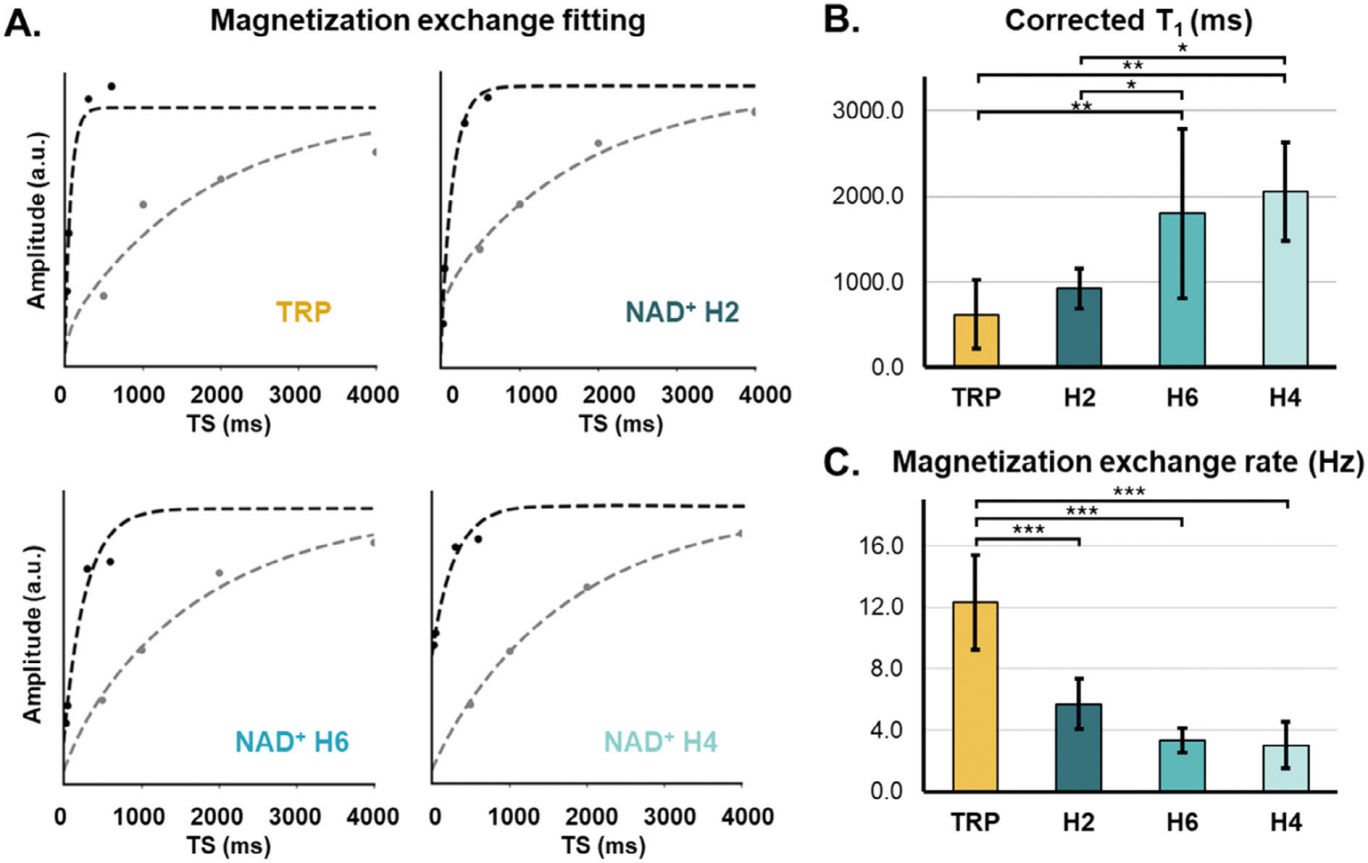
(A) Representative selective (black) and broadband (gray) saturation T_1_ recovery curves of the tryptophan (TRP) and NAD^+^ H2, H6, and H4 resonances after simultaneous fitting with a model for a two‐spin system (Model 2). The last saturation time (TS = 10 s assigned to the M_0_ scan) is not shown for illustrative purposes. (B) Mean T1 corrected for magnetization exchange and (C) magnetization exchange rate determined for tryptophan and NAD^+^. *p < 0.05, **p < 0.01, ***p < 0.001.

We additionally modeled the T_1_ relaxation time, corrected for magnetization exchange effects, and the magnetization exchange rate using the modified Bloch equations (Equations 2 and 3), simultaneously fitting the selective and broadband saturation recovery curve data (Figure 4A). The smallest *R* ^2^ of the simultaneous fit was 0.90, and the average *R* ^2^ was 0.98 ± 0.02. The corrected T_1_ relaxation times were: T_1_,_TRP_ = 622.3 ± 405.5 ms; T_1,NAD,H2_ = 924.9 ± 233.7 ms; T_1,NAD,H6_ = 1800.0 ± 985.5 ms; T_1,NAD,H4_ = 2057.3 ± 573.5 ms (Table 1). We performed ANOVA, which showed there was a significant difference in T_1_ across the three peaks (*p* < 0.001), and post hoc tests that revealed that the H6 proton of NAD^+^ had a significantly longer T_1_ than NAD^+^ H2 or tryptophan (NAD^+^ H6 vs. TRP: *p* = 0.007; NAD^+^ H6 vs. NAD^+^ H2: *p* = 0.047), as did the H4 proton of NAD^+^ (NAD^+^ H4 vs. TRP: *p* = 0.001; NAD^+^ H4 vs. NAD^+^ H2: *p* = 0.010) (Figure 4B). The cross‐relaxation rates fit by modeling the two saturation recovery experiments simultaneously were: *σ*_TRP_ = 12.3 ± 3.1 Hz; *σ*_NAD,H2_ = 5.7 ± 1.6 Hz; *σ*_NAD,H6_ = 3.3 ± 0.8 Hz; *σ*_NAD,H4_ = 3.0 ± 1.5 Hz (Table 1). The magnetization exchange rate was significantly different between the resonances (*p* < 0.001), and post hoc tests showed that specifically, the magnetization exchange rate of tryptophan was significantly higher than each of the NAD^+^ resonances (TRP vs. NAD^+^ H2: *p* < 0.001; TRP vs. NAD^+^ H6: *p* < 0.001; TRP vs. NAD^+^ H4: *p* < 0.001) (Figure 4C).

**TABLE 1:** Two‐spin model fits of T_1_ corrected for exchange effects and of magnetization exchange rate of tryptophan (TRP) and NAD^+^ in all subjects.

Subject #	T_1_ corrected for magnetization exchange (ms)				Magnetization exchange rate (Hz)			
	TRP	NAD^+^ H2	NAD^+^ H6	NAD^+^ H4	TRP	NAD^+^ H2	NAD^+^ H6	NAD^+^ H4
1	1374.7	1308.9	1365.9	1415.2	15.3	5.0	4.9	2.2
2	452.8	647.6	2562.9	2761.3	8.2	6.3	2.7	1.2
3	662.0	1038.0	997.0	1796.5	9.2	7.4	2.6	2.1
4	251.8	926.6	1539.0	1520.0	12.7	6.1	3.7	2.9
5	759.5	1025.3	1329.0	2192.2	12.1	6.0	2.9	4.8
6	146.2	594.0	NA	NA	16.9	6.2	NA	NA
7	NA	816.6	1100.4	2658.6	NA	6.7	3.6	4.9
8	709.2	1042.3	3706.1	NA	12.0	2.0	2.9	NA
Mean ± SD	622.3 ± 405.5	924.9 ± 233.7	1800.0 ± 985.5	2057.3 ± 573.5	12.3 ± 3.1	5.7 ± 1.6	3.3 ± 0.8	3.0 ± 1.5

## 4 Discussion

In this work, we quantified the effect of magnetization exchange on longitudinal magnetization recovery and measured magnetization exchange rates for tryptophan and NAD^+^ resonances in the downfield region of the proton spectrum at 7 T in the human brain in vivo. While magnetization exchange has been investigated for multiple upfield and downfield metabolites [5, 6, 27], it has not been characterized for resonances further downfield in the proton MR spectrum that arise from lower concentration metabolites. Our results indicate that there are significant differences in magnetization exchange between metabolites that resonate downfield of 8.5 ppm. The metabolites we investigated in this work were tryptophan, which exhibits a singlet resonance at 10.1 ppm, and NAD^+^, which exhibits three resonances downfield of 8.5 ppm (a singlet at 9.3 ppm and two doublets at 9.1 and 8.9 ppm). In order to target both metabolites, the spectrally selective pulses were centered equally between the 10.1 ppm tryptophan resonance and the 9.3 ppm NAD^+^ resonance. Because the avoidance of water excitation is critical to the detection of these downfield metabolites and the observation of their magnetization exchange effects, the spectrally selective excitation and saturation pulses were kept limited to a bandwidth of 2 ppm [10]. However, it should be noted that the 8.9 ppm resonance of NAD^+^ lies on the transition band of the pulse and overlaps with the tail of the complex of resonances directly upfield of NAD^+^. This complex arises from adenosine, NAA, and amide protons in peptides/proteins. These additional resonances are also partially excited at the transition band of the selective sinc pulse, which complicates the fitting of the 8.9 ppm resonance and may account for the poor fit quality for the NAD^+^ H4 proton seen in two subjects.

We first measured the effect of magnetization exchange using a spectrally selective sequence in a paired selective and broadband saturation recovery experiment. We first fit a simple three‐parameter model of longitudinal recovery (Model 1), as is commonly done in T_1_ experiments, in order to measure an “apparent T_1_” for each saturation recovery experiment independently. This apparent T_1_ implicitly includes the effects of magnetization exchange between the metabolites and water. For tryptophan, we measured a stronger effect of magnetization exchange on longitudinal relaxation than for NAD^+^, with the broadband apparent T_1_ being 19 times longer than the selective apparent T_1_ for tryptophan, compared to an average of approximately 9 times longer across all three NAD^+^ resonances. This increase in apparent T_1_ relaxation time for NAD^+^ was slightly larger than was observed in rat brain in situ at 11.7 T, which showed approximately a four‐fold increase in T_1_ relaxation measured using nonselective and selective inversion recovery with a localization by adiabatic spin‐echo refocusing (LASER) sequence [2]. We note, though, that these values cannot be directly compared due to differences in field strength, sequences used, organism, and experimental conditions (i.e., in vivo vs. in situ). Both NAD^+^ and tryptophan also showed a greater increase in longitudinal relaxation in human brain in vivo compared to two unassigned downfield peaks resonating at approximately 7.9 and 8.2 ppm measured by Shemesh et al. in ex vivo mouse brains at 9.4 T, where using water suppression led to T_1_ relaxation times that were 3 times longer than without water suppression [6].

In order to explicitly model the rate of magnetization exchange between the metabolites and water, we additionally fit a model for two spins consisting of coupled differential equations (Model 2). The model included a term for the T_1_ relaxation rate corrected for exchange, as well as terms for the forward and backward magnetization exchange between the two spin pools [36, 37]. In this model, the saturation efficiency was not left as a free parameter but rather used the modeled saturation efficiencies from the apparent T_1_ fitting scheme. The optimization procedure utilized a basin‐hopping approach in order to avoid finding local minima. We found that the NAD^+^ H6 and H4 protons had a fitted T_1_ relaxation time that was significantly longer than tryptophan or the NAD^+^ H2. This is consistent with the NAD^+^ H6 and H4 protons having longer apparent NAD^+^ T_1_ relaxation times than the H2 proton, which has been regularly observed, as well as having smaller increases in apparent T_1_ between non‐water‐perturbed and water‐perturbed experiments. Supporting the findings on apparent T_1_ relaxation measurements, we also found the magnetization exchange for tryptophan to be more rapid at 12.3 Hz than for all NAD^+^ resonances, which ranged from 3.0 to 5.7 Hz. The average cross‐relaxation rates for NAD^+^ in the brain measured in this study fall between previously determined cross‐relaxation rates of carnosine (0.8 Hz) and adenosine (6.6 Hz) in vivo in human calf muscle [7]. Other magnetization exchange rates for downfield resonances measured by MacMillan et al. in the human brain at 3 T ranged from approximately 0.5–8 Hz [5]. In agreement with the indication from apparent T_1_ measurements that downfield metabolites have larger magnetization exchange effects than their non‐labile upfield counterparts on the timescale of these experiments, the quantified magnetization exchange rates of tryptophan and NAD^+^ also appear to be generally larger—creatine, which has one of the strongest upfield magnetization exchange effects, was measured to have an exchange rate of only 0.3 and 0.3–0.6 Hz in rat brain [42] and human calf muscle [27], respectively.

One reason for the difference between the magnetization exchange rates of tryptophan and NAD^+^ may be related to the method of magnetization exchange for the two metabolites. The tryptophan proton that resonates at 10.1 ppm is part of the amine group (NH) of the indole moiety in tryptophan, and this proton undergoes chemical exchange with water [43, 44]. The NAD^+^ resonances are non‐labile protons on the nicotinamide moiety and thus undergo cross‐relaxation with water [2]. This is likely a contributing factor to the broadness of the tryptophan peak in comparison to the NAD^+^ peaks. While chemical exchange rates are pH and temperature‐dependent, cross‐relaxation rates rely on mechanisms that affect the molecular correlation time, such as binding to large macromolecules, since cross‐relaxation is a dipole–dipole interaction with a dependence on 1/*r* ^6^ [37], where *r* is the distance between the two protons. These different biological origins of magnetization exchange may give rise to the different magnetization exchange rates observed between metabolites [45]. Further experiments can help elucidate the exact mechanism of cross‐relaxation for NAD^+^, as there are multiple pathways available for non‐labile proton magnetization exchange, some of which may be mediated through chemical exchange between labile protons of NAD^+^ and water. The quantification of a chemical exchange rate for tryptophan and cross‐relaxation rate for NAD^+^ will aid in future protocol design and quantification of these metabolites. Additionally, these exchange rates may be biomarkers of changing microenvironments in different diseases that affect pH or metabolite binding. While the tryptophan proton shows a CEST effect at higher concentrations in vitro [46], it remains to be determined if such an effect at the lower physiological concentration (< 0.2 mM) is within the sensitivity limit of CEST in vivo.

There are several limitations in this study. Firstly, the saturation RF pulse is susceptible to B_1_ inhomogeneities, particularly at the ultra‐high field strength, resulting in reduced saturation efficiency. This inefficiency is accounted for by incorporating a saturation efficiency parameter into the T_1_ and magnetization exchange models, though there is an additional limitation in that the saturation efficiency may vary across the large volume selected. While adiabatic saturation pulses may overcome this limitation, these pulses typically have a long duration and higher energy deposition, which limit their use for downfield spectroscopy, which targets short T_2_ species, particularly at high field. Nonetheless, in our study, all resonances had a saturation efficiency above 0.7 except for selective saturation of the NAD^+^ H4 proton, which was to be expected due to its proximity to the transition band of the pulse. Additionally, due to the lengthy experiment time, only 64 averages were collected for the saturation recovery experiment spectra, resulting in an SNR too low in some subjects to be able to adequately fit the tryptophan resonance and leading to a reduction in the sample size. Furthermore, though the tryptophan peak is well‐isolated, the NAD^+^ resonances lie on the shoulder of a complex of resonances whose exact composition remains unknown. Because there is some saturation and excitation of this complex that occurs, there may be additional contributions from these resonances that appear in the NAD^+^ peaks. We used an HSVD approach for peak fitting in order to fit these extraneous resonances and remove them from the downfield region of interest. While a basis‐set approach for fitting, like LCModel, may be used to fit NAD^+^, it would require prior knowledge of these overlapping peaks, which is not currently available. Future studies may optimize the saturation scheme to enable acquisition of an increased number of averages or choose to focus on a more limited number of resonances (e.g., only the H2 proton of NAD^+^) in order to increase robustness and reliability of the measuring magnetization exchange. A further limitation in this study is the large slice volume used. Due to this, we could not determine region or tissue‐specific magnetization exchange effects. Chemical shift imaging allows the spatial resolution of downfield metabolite maps, but these methods typically require lengthy acquisitions (though further improvements such as EPSI‐based encoding may reduce acquisition time) and have yet to exhibit sufficient SNR to detect NAD^+^ and tryptophan [47, 48, 49]. A spatially resolved method would also benefit from the ability to correct for spatial B_1_ variations with B_1_ mapping. Additionally, further improvements could be made in the assumptions used in the two‐spin model, where a single value for the T_1_ relaxation time of water was assumed, though the slice contains gray matter, white matter, and cerebrospinal fluid.

## 5 Conclusion

Downfield metabolites may chemically exchange or cross‐relax with water in the human brain in vivo. The effect of these magnetization exchange mechanisms was observed through the shortening of apparent T_1_ relaxation times for tryptophan and NAD^+^ under conditions without water perturbation. The magnetization exchange rate was quantified in vivo in using downfield spectroscopy, and the chemical exchange rate of tryptophan was found to be faster than the cross‐relaxation rate of NAD^+^. Quantification of magnetization exchange allows further characterization and exploitation of the relaxation properties of these metabolites.

## Supporting information


**Table S1:** Minimum reporting standards for in vivo MRS.
**Table S2:** Apparent T_1_ relaxation times measured with selective and broadband saturation of tryptophan (TRP) and NAD^+^.
**Figure S1:** Representative selective (left) and broadband (right) saturation recovery spectra at varying saturation times (TS) from a single subject.

## Data Availability

The data that support the findings of this study are available from the corresponding author upon reasonable request. The code developed to analyze the acquired data are available at https://github.com/markymarkymark/SpecTickle.
