# Supplementary material for: Quantification of Tryptophan and NAD + Proton Magnetization Exchange With Water Using Downfield 1H MRS in the Human Brain at 7 T
Source: Magn Reson Med. 2025 Oct 14;95(3):1336–44. doi: 10.1002/mrm.70134 (PMC12746366; doi:10.1002/mrm.70134)
Supplement: Supplementary file 1 — Table S1: Minimum reporting standards for in vivo MRS. Table S2: Apparent T1 relaxation times measured with selective and broadband saturation of tryptophan (TRP) and NAD+. Figure S1: Representative selective (left) and broadband (right) saturation recovery spectra at varying saturation times (TS) from a single subject. [file MRM-95-1336-s001.docx]

**Supporting Information**

**Supporting Information Table S1.** Minimum Reporting Standards for in vivo MRS

| 1. Hardware |  |
| --- | --- |
| a. Field strength [T] | 7 T |
| b. Manufacturer | Siemens |
| c. Model (software version if available) | Terra (VE12u) |
| d. RF coils: nuclei (transmit/receive), number of channels, type, body part | 1 Tx / 32 Rx head coil (Nova Medical) |
| e. Additional hardware | N/A |
| 2. Acquisition |  |
| a. Pulse sequence | Single-slice spectrally-selective spectroscopy |
| b. Volume of Interest (VOI) locations | Obliqued axial brain slice |
| c. Nominal VOI size [cm^3^, mm^3^] | 390-530 cm^3^ |
| d. Repetition Time (TR), Echo Time (TE) [ms, s] | Downfield selective saturation experiment: TR 1200 ms; TE 13 ms, TS: 25, 50, 300, 600 ms  Downfield broadband saturation experiment: TR 6500 ms; TE 13 ms, TS: 500, 1000, 2000, 4000 ms  Downfield non-saturation scan: TR: 2000 ms, TE: 13 ms  Water reference scan: TR: 10 s; TE: 13 ms |
| e. Total number of Excitations or acquisitions per spectrum | Saturation recovery spectra: NEX = 64  Non-saturation spectrum: NEX = 128  Water reference spectrum: NEX = 16 |
| f. Additional sequence parameters (spectral width in Hz, number of spectral points, frequency offsets) | Spectral excitation and selective saturation center: 9.7 ppm, bandwidth 2 ppm  Broadband saturation center: 7.4 ppm  Water reference excitation center: 4.7 ppm  Readout bandwidth 4000 Hz  2048 spectral points |
| g. Water Suppression Method | N/A |
| h. Shimming Method, reference peak, and thresholds for “acceptance of shim” chosen | Automatic brain shimming followed by manual shimming to water linewidth < 25 Hz |
| i. Triggering or motion correction method | N/A |
| 3. Data analysis methods and outputs |  |
| a. Analysis software | Matlab: data pre-processing and analysis  <https://github.com/markymarkymark/SpecTickle> |
| b. Processing steps deviating from quoted reference or product | N/A |
| c. Output measure  (e.g., absolute concentration, institutional units, ratio) | Apparent T_1_ relaxation time  T_1_ relaxation time in absence of magnetization exchange  Magnetization exchange rate |
| d. Quantification references and assumptions, fitting model assumptions | Assumptions for 2-spin fitting model: water T_1_ = 1800 ms, metabolite to water ratio M_z,B,0_/M_z,A,0_ =0.3 mM/(2*55) M |
| 4. Data Quality |  |
| a. Reported variables  (SNR, Linewidth (with reference peaks)) | Water linewidth  Metabolite linewidth |
| b. Data exclusion criteria | Peak fitting failure |
| c. Quality measures of postprocessing Model fitting (e.g. CRLB, goodness of fit, SD of residual) | Goodness of fit (R^2^) of T_1_ and magnetization exchange fitting |
| d. Sample Spectrum | Figure 2 |

**Supporting Information Table S2.** Apparent T_1_ relaxation times measured with selective and broadband saturation of tryptophan (TRP) and NAD^+^.

|  | **Broadband apparent T_1_ (ms)** | **Broadband saturation efficiency** | **Selective apparent T_1_ (ms)** | **Selective saturation efficiency** | **Ratio of broadband:selective apparent T_1_** |
| --- | --- | --- | --- | --- | --- |
| **TRP**  **(n=7)** | 1304.6±236.0 | 0.90±0.09 | 72.8±21.6 | 0.84±0.19 | 19.3±6.2 |
| **NAD^+^ H2 (n=8)** | 1573.3±295.0 | 0.79±0.05 | 139.5±19.6 | 0.94±0.06 | 11.2±1.5 |
| **NAD^+^ H6 (n=7)** | 1808.8±423.1 | 0.81±0.05 | 228.7±44.2 | 0.72±0.05 | 7.6±2.3 |
| **NAD^+^ H4 (n=6)** | 1860.5±213.3 | 0.80±0.10 | 329.3±149.8 | 0.42±0.10 | 6.4±2.4 |


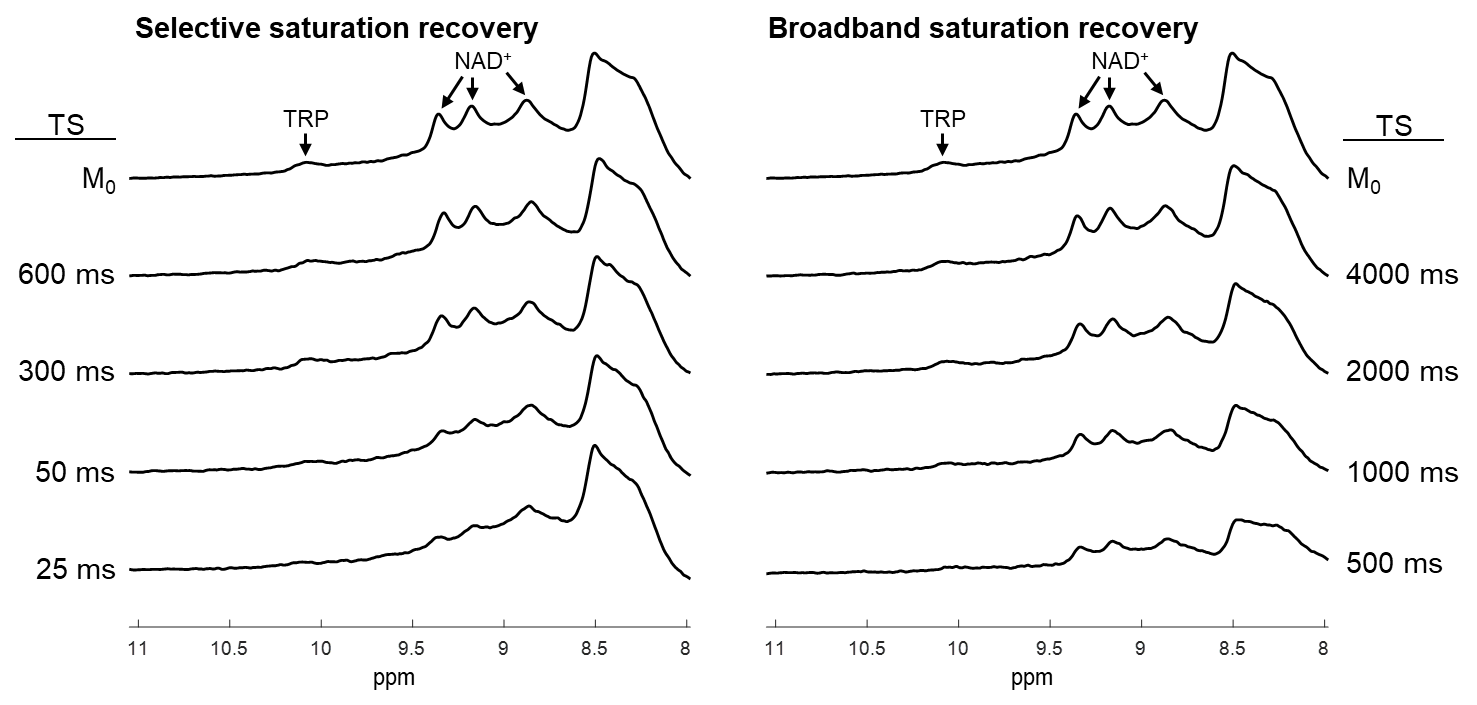


**Supporting Information Figure S1:** Representative selective (left) and broadband (right) saturation recovery spectra at varying saturation times (TS) from a single subject.
